# Transomental hernia causing small bowel obstruction: an uncommon surgical emergency and literature review

**DOI:** 10.1093/jscr/rjaf670

**Published:** 2025-08-29

**Authors:** Mohammed N AlAli, Jawad S Alnajjar, Mohamed S Essa, Arwa F Alrasheed, Laiba Yaseen, Nouf A Alromaih, Ruba M Alzuhairi, Ossama AlAmri

**Affiliations:** Department of Surgery, Prince Mohammed Bin Abdulaziz Hospital, Ministry of Health, Riyadh, Saudi Arabia; College of Medicine, King Faisal University, Alahsa, Saudi Arabia; Department of Surgery, Prince Mohammed Bin Abdulaziz Hospital, Ministry of Health, Riyadh, Saudi Arabia; General Surgery Department, Faculty of Medicine, Benha University, Benha Egypt; Department of Surgery, Prince Mohammed Bin Abdulaziz Hospital, Ministry of Health, Riyadh, Saudi Arabia; College of Medicine, Alfaisal University, Riyadh, Saudi Arabia; Department of Surgery, Prince Mohammed Bin Abdulaziz Hospital, Ministry of Health, Riyadh, Saudi Arabia; Department of Surgery, Prince Mohammed Bin Abdulaziz Hospital, Ministry of Health, Riyadh, Saudi Arabia; Department of Surgery, Prince Mohammed Bin Abdulaziz Hospital, Ministry of Health, Riyadh, Saudi Arabia

**Keywords:** transomental hernia, internal hernia, small bowel obstruction, abdominal CT, surgical emergency, omental defect

## Abstract

Transomental hernia (TOH) is an exceedingly rare subtype of internal hernia, often leading to acute small bowel obstruction with a high risk of ischemia. We present the case of a 58-year-old male with a history of laparoscopic inguinal hernia repair who developed abdominal pain, distension, vomiting, and obstipation. Contrast-enhanced computed tomography revealed clustered dilated ileal loops with a transition point suggestive of internal herniation. Exploratory laparotomy confirmed a TOH through the greater omentum without bowel ischemia. The defect was divided, and the postoperative course was uneventful. Due to its nonspecific presentation and diagnostic difficulty, TOH is frequently identified only at surgery. Prompt recognition and timely surgical management are essential to prevent serious complications.

## Introduction

An internal hernia (IH) refers to the protrusion of abdominal viscera through congenital or acquired defects within peritoneal cavity, involving structures such as mesentery or omentum. Though infrequent, IH is responsible for ⁓0.5%–3% of all cases of intestinal obstruction and carries a notable risk of morbidity and mortality [1]. The most frequently encountered subtypes include paraduodenal (53%), pericecal (13%), foramen of Winslow (8%), transmesenteric (8%), pelvic (7%), transmesosigmoid (6%), with less common forms comprising the remaining 5%.

Transomental hernia (TOH) is among the rarest subtypes, accounting for ⁓2% of all IHs, and is particularly challenging to identify before surgery [1, 2]. Here, we describe a case involving an adult male with a history of abdominal surgery who presented with clinical features of acute small bowel obstruction. Preoperative imaging suggested an IH, although its specific type was not evident until intraoperative exploration revealed a TOH.

## Case presentation

A 58-year-old male with a medical history of hypertension and diabetes mellitus, as well as a prior laparoscopic inguinal hernia repair, presented to the emergency department with a 3-day history of abdominal pain, distension, vomiting, and obstipation. He denied any previous episodes of similar symptoms. On physical examination, the patient was conscious, alert, and tachycardic, but remained hemodynamically stable. Abdominal examination revealed generalized distension with localized tenderness, most prominent in the left upper quadrant, and no signs suggestive of generalized peritonitis. Digital rectal examination showed an empty rectum and was otherwise unremarkable. Laboratory investigations demonstrated leukocytosis (white blood cell: 14.9 × 10^9^/L), with a normal platelet count (194 × 10^9^/L) and serum lactate level (1.9 mmol/L). Other laboratory values were within normal limits.

**Figure 1:**
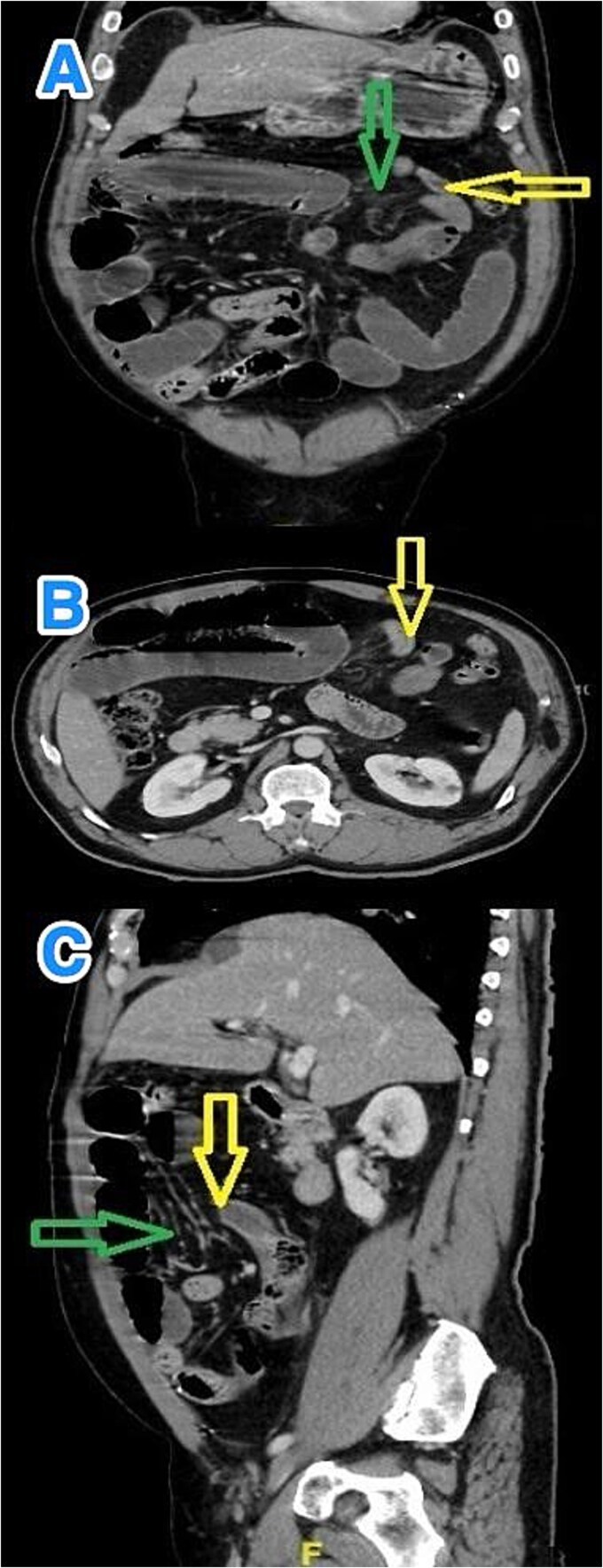
Multiview contrast-enhanced CT images of the abdomen demonstrate: (A) a coronal view showing herniated bowel (yellow arrow) through the greater omentum (green arrow), (B) an axial view showing the transition zone (yellow arrow), and (C) a sagittal view showing herniated bowel (yellow arrow) through the greater omentum (green arrow).

Contrast-enhanced abdominal computed tomography (CT) revealed multiple dilated small bowel loops, measuring up to 3.8 cm in diameter, along with air-fluid levels. The terminal ileum appeared elongated, and distal ileal loops were clustered in the left upper quadrant. A transition point was identified just proximal to these loops, raising suspicion for an IH rather than an adhesive obstruction, and signs of bowel fatigue were present. No free intraperitoneal air, lymphadenopathy, fluid collections, or peritoneal implants were noted (Fig. 1).

**Figure 2:**
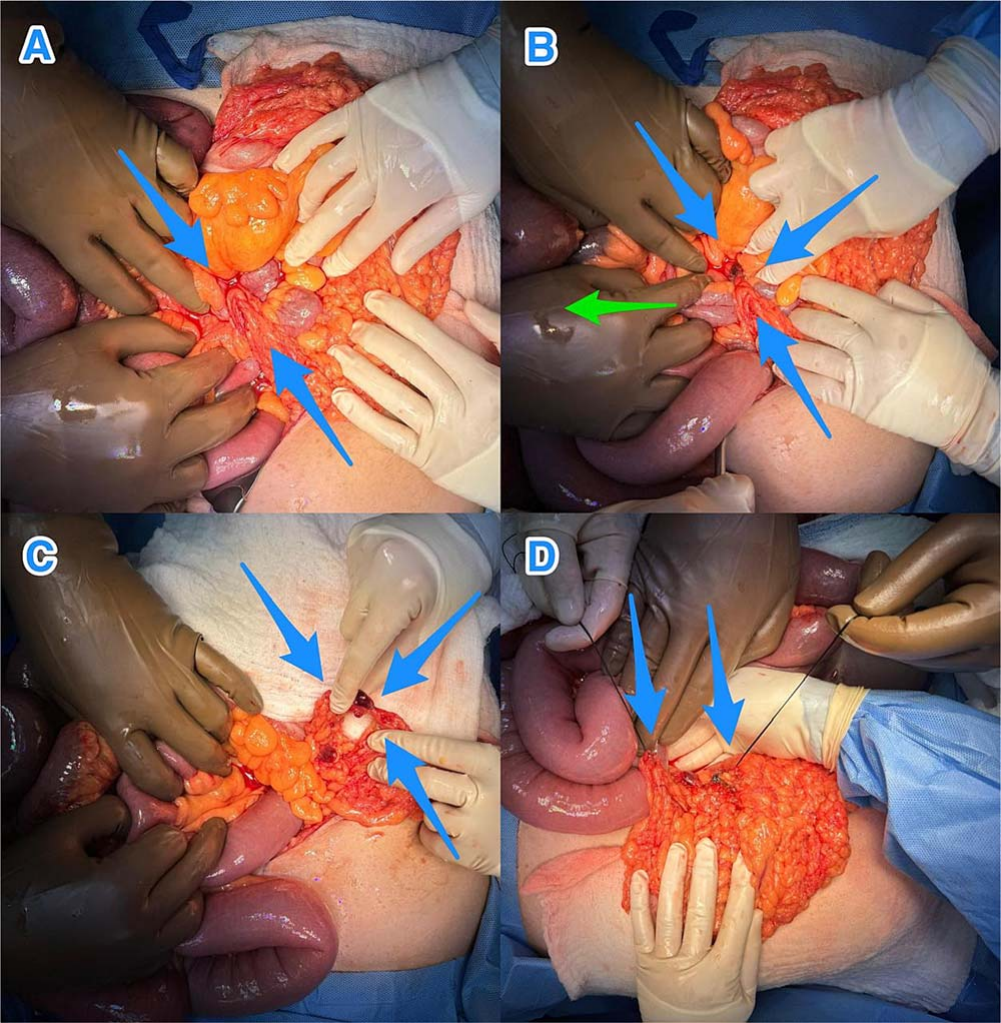
Intraoperative views revealing: (A) a small bowel loop herniating through an omental defect of the greater omentum (blue arrows), (B) milking of the small bowel loops (green arrow) to reduce the herniated loop through the defect (blue arrows), (C) the transomental defect after reduction (blue arrows), and (D) the dividing omental defect (blue arrows).

Following initial resuscitation and nasogastric decompression, the patient underwent exploratory laparotomy. Intraoperative assessment revealed herniation of a proximal ileal loop through a defect in the greater omentum—confirming a TOH. The herniated bowel was reduced, with no evidence of ischemia or necrosis. The omental defect was surgically divided (Fig. 2), and a thorough inspection ruled out other intra-abdominal abnormalities such as adhesions. The postoperative course was uneventful. At the most recent follow-up, ⁓3 months after surgery, the patient remained asymptomatic and in good health.

## Discussion

TOH is one of the rarest forms of IH, accounting for ⁓2% of cases. It is generally acquired, often linked to previous abdominal surgeries—especially procedures like Roux-en-Y gastric bypass that involve omental manipulation. Nevertheless, spontaneous TOH can occur in patients without surgical history, typically due to age-related omental atrophy [3, 4]. Other contributing factors include elevated intra-abdominal pressure (e.g. from chronic cough, ascites, or obesity), prior intra-abdominal inflammation, trauma, and congenital connective tissue abnormalities [5, 6]. In the present case, the patient's history of inguinal hernia repair may have been a predisposing factor.

Diagnosing TOH before surgery remains difficult due to its nonspecific presentation and absence of a hernia sac, which is a hallmark of other IHs. The typically small hernia orifice increases the likelihood of strangulation and bowel ischemia [7]. Most cases are identified intraoperatively, often after delayed presentation, which can result in necrosis and carries a postoperative mortality rate of over 30% [6, 8].

CT is the preferred diagnostic tool for IHs, including TOH. However, its accuracy in identifying TOH is limited by the hernia’s rarity and often subtle or transient imaging features [4]. In a study involving 49 surgically confirmed IHs, only 16% were diagnosed preoperatively on CT scans [9]. Characteristic findings may include clustered dilated small bowel loops, air-fluid levels, a transition point or "beak sign," and twisted mesenteric vessels indicating obstruction or ischemia [10]. A particularly specific finding for TOH is the posterior displacement of the transverse colon relative to dilated bowel loops, with reported sensitivity and specificity of 71% and 94%, respectively [11]. Additional CT features such as convergence of mesenteric vessels, swirling of omental fat, and absence of interposed fat planes may support the diagnosis, though differentiating TOH from other hernias or volvulus remains challenging [7, 12].

Surgical intervention is the definitive treatment for TOH. Exploratory laparotomy remains the gold standard in emergent or uncertain cases, providing optimal exposure for diagnosis, bowel evaluation, and resection if needed [4]. Laparoscopy may be an option in stable patients without signs of ischemia, though it limits direct assessment of bowel viability. In such cases, indocyanine green fluorescence imaging may enhance evaluation of intestinal perfusion [13–15]. If ischemic bowel is encountered, resection and anastomosis are essential. The omental defect should be closed or divided to prevent recurrence, and partial omentectomy may further reduce risk [5].

In this case, open surgery enabled prompt reduction of the hernia and division of the omental defect without the need for bowel resection. The patient's recovery was uneventful. Timely diagnosis and early surgical management are vital to reduce the risk of serious complications such as bowel infarction and sepsis [3, 4].

## Conclusions

TOH is a rare and difficult-to-diagnose cause of bowel obstruction, often requiring surgical confirmation. While commonly linked to prior surgeries, it can occur spontaneously. Early recognition and prompt surgical management are vital to avoid serious complications, including bowel ischemia, necrosis, and increased morbidity or mortality.
